# Fatigue Modeling Containing Hardening Particles and Grain Orientation for Aluminum Alloy FSW Joints

**DOI:** 10.3390/ma12122024

**Published:** 2019-06-24

**Authors:** Guoqin Sun, Yicheng Guo, Xiuquan Han, Deguang Shang, Shujun Chen

**Affiliations:** 1 College of Mechanical Engineering and Applied Electronics Technology, Beijing University of Technology, Beijing 100124, China; guoyicheng@emails.bjut.edu.cn (Y.G.); shangdg@bjut.edu.cn (D.S.); sjchen@bjut.edu.cn (S.C.); 2 ACIV Manufacturing Technology Institute, Beijing 100024, China; james_hxq@sina.com

**Keywords:** friction stir welded joint, strengthening particles, crystal plasticity theory, stress and strain responses

## Abstract

The macro-mesoscopic joint fatigue model containing hardening particles and crystal characteristics is established to study the effect of the hardening particles and the grain orientation on fatigue properties of an aluminum alloy friction stir welding (FSW) joint. The macroscopic model is composed of the weld nugget zone, thermo-mechanically affected zone, heat-affected zone, and base material, according to the metallurgical morphology and hardness distribution of the joint. Cyclic stress and strain data are used to determine the material properties. The fatigue parameters used in the calculation of cyclic stresses and strains are obtained with the four-point correlation method. The mesoscopic models of different zones are inserted into the joint macroscopic model as submodules. The models containing the information of hardening particles and grain orientation are established with crystal plasticity theory for the grains and isotropic hardening rule for the hardening particles. The effects of hardening particles and grain orientation on the stress and strain responses are discussed. The simulation results show that high-angle misorientation of adjacent grains hinders the stress transfer. The particle cluster or cracked particles intensify the stress and strain concentrations.

## 1. Introduction

Friction stir welding (FSW), as a solid-state joining technique, has been found to be effective for joining high strength alloys of the 2xxx and 7xxx series, which is extensively used in the aircraft industry. Analysis of fatigue properties and prevention of fatigue failure for the welded structure is a major issue for the aircraft industry. Some experimental analysis and numerical simulation have been made in the fundamental understanding of the properties of the FSW joints.

Static mechanical properties of friction stir welded (FSW) joint have been found to be affected by a local microstructure of the joint [1,2,3,4,5,6,7]. Research on the relation between microstructure evolution and mechanical properties in the FSW joint, grain size, grain orientation, and texture variation are taken into account. The finer grain size, the higher angle grain boundaries and more random textures in the stir zone lead to the higher strength and larger elongation of the joint [3,4,5]. The changes of grain orientation and texture have an effect on the mechanical properties [6]. Falla et al. [5] found that lower hardness and larger grains in the heat-affected zone (HAZ) increased the sensitivity to cracking in the HAZ.

Fatigue behavior and fracture of the joints are sensitive to the microstructures of different zones for the joints [8]. Besel et al. [9] found that fatigue crack initiation and the following propagation of FSW aluminum Al-Mg-Sc alloy were influenced by microstructure and hardness distribution. Crack initiation behavior and fatigue life are attributed to the local high plastic straining behavior and the heterogeneous microstructure. Masoumi et al. [10] considered that grains with a high Taylor factor and kernel average misorientation are prone to fatigue crack propagation. Wu et al. [11] found that small cracks tended to initiate at inclusion-particle clusters, grain boundaries, slip bands, and voids. Sun et al. [12] found that most of the cracks were prone to initiate around some hardening particles in 7075 FSW joints fatigue tests. The fatigue cracks initiated easily around the second phase particles in aluminum alloy [12,13,14]. Hard constituent particle cracking was observed to be the sole cause for fatigue crack initiation in the 7075-T651 alloy [13].

The mechanical behavior of FSW joints can be simulated to obtain the local stresses and strains and the weak areas, which are beneficial to the property analysis and life evaluation for the components. Lockwood et al. [15] simulated the local mechanical response of FSW AA2024 with local constitutive data input in the various weld regions. Simar et al. [16] modelled the tensile behavior using the local tensile properties measured with the micro-tensile specimens extracted from different regions in the joint. A computational study on the effect of polycrystalline microstructure on the plastic strain localization and fracture of FSW aluminum alloys under mechanical loading shows that they are determined by the microstructure of materials in the advancing side of the weld [17]. Crystal plasticity simulation on a volume of material containing several textured bands shows the heterogeneity of the strain field [18]. Fatigue behavior and weak areas of the FSW joint were simulated with an FSW joint model including the characteristics of hardening phases [19]. The local mechanical response and fatigue life prediction of 2219 FSW joints were proceeded based on crystal plasticity modeling for the FSW joint [20].

To study the effect of hardening particles and crystal characteristics on the local mechanical properties and fatigue weak areas of aluminum alloy FSW joints, a macro-mesoscopic FSW joint model containing microstructure and macro local properties of different areas is built. The effects of hardening particles and grain orientation on the responses of local stress and strain are discussed to study the effect of microstructure on the mechanical properties of the joint.

## 2. Materials and Methods

### 2.1. Experiments

**Figure 1:**
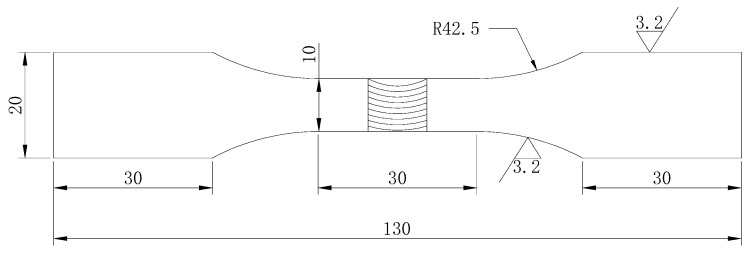
Shape and size of the fatigue specimen.

The specimens were cut from FSW butt welded plates 300 mm × 300 mm × 6mm of 2219-T6 aluminum alloy (CALT, Beijing, China), as shown in Figure 1. The welding parameters had a rotation speed of 800 rpm and a forward speed of 150mm/min. The welding direction was perpendicular to the rolling direction.

**Figure 2:**
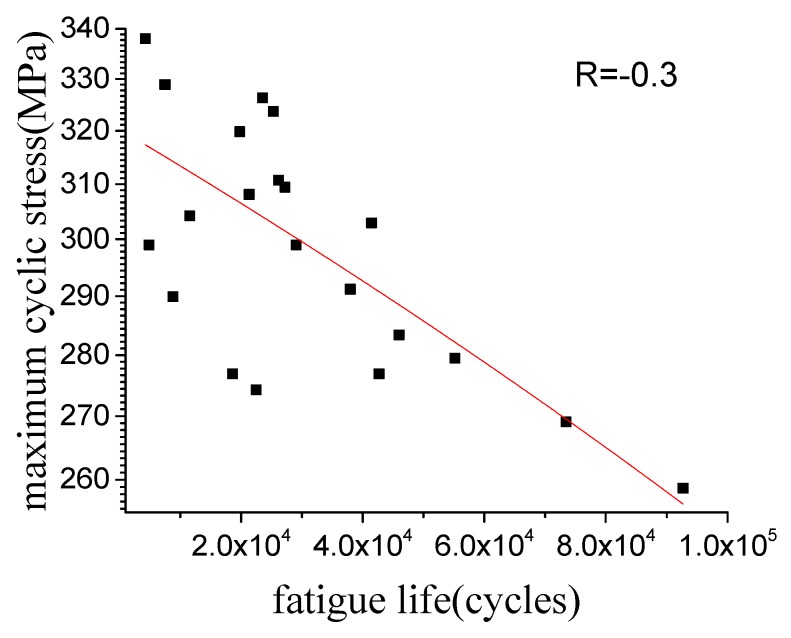
Fatigue curve of the FSW joint.

The fatigue tests with a stress ratio of −0.3 were conducted using MTS-858 hydraulic servo system (MTS Systems, Minneapolis, MN, USA) with a frequency of 10 Hz. The loading direction for the specimen was perpendicular to the weld line. The detailed fatigue data can be found in the literature [19]. The curve of maximum stress versus fatigue life is shown in Figure 2.

### 2.2. Fatigue Fracture Analysis

**Figure 3:**
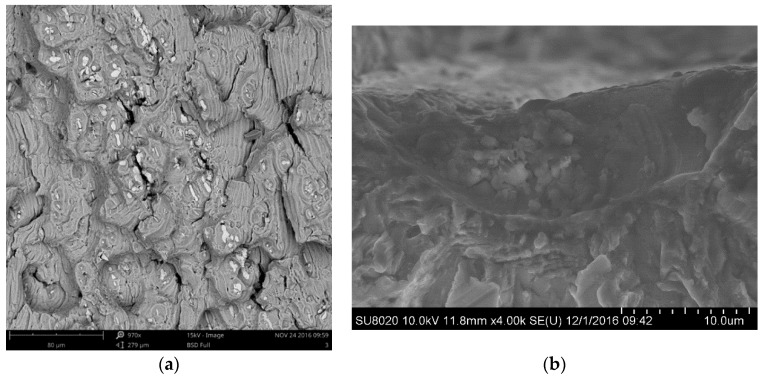
Morphologies of the (**a**) crack propagation area and (**b**) crack initiation site.

The fracture morphology is observed with Phenom Pro X (FEI, Hillsboro, OR, USA) and Hitachi SU 8020 scanning electron microscopes (Hitachi, Tokyo, Japan). Some secondary cracks around hardening particles were found in the region of multiparticle aggregation, as shown in Figure 3a. Secondary cracks near the hardening particles extend along the boundary between the hardening particles and the matrix. The main crack initiation site has an affinity with the particle cluster, as shown in Figure 3b. The cracked hardening particles and particle cluster are prone to cause the crack initiation and growth.

### 2.3. Crystal Morphologies

**Figure 4:**
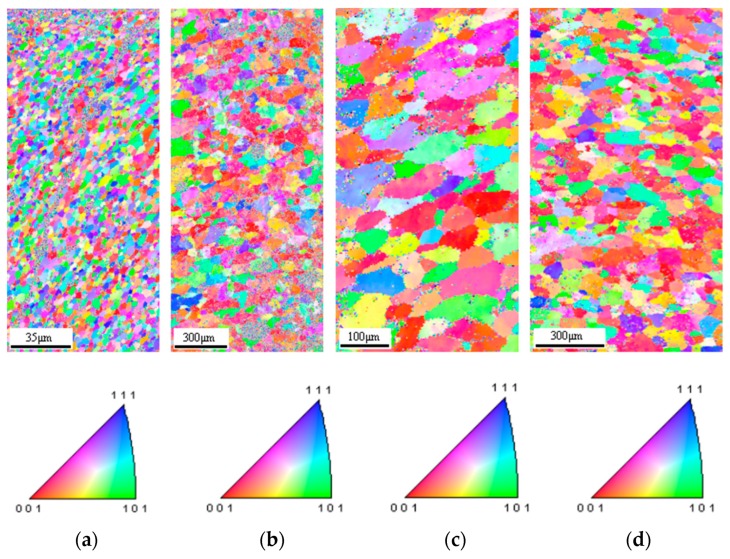
Crystal morphology distribution in the (**a**) WNZ, (**b**) TMAZ, (**c**) HAZ, and (**d**) BM of the joint.

**Figure 5:**
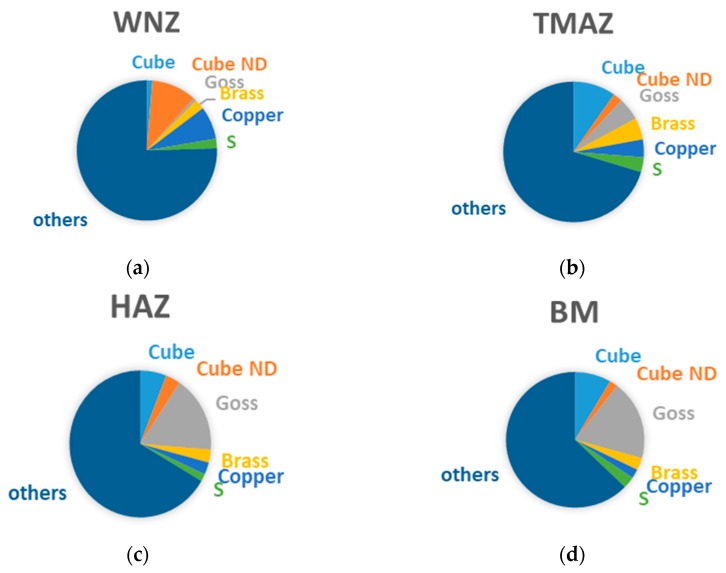
Texture distribution in the (**a**) WNZ, (**b**) TMAZ, (**c**) HAZ, and (**d**) BM of the joint.

The crystal morphologies in the joint are shown in Figure 4, which are obtained with JSM-6500F field emission scanning electron microscope (SEM, JEOL, Tokyo, Japan). The diameters of most particles in the weld nugget zone (WNZ) are about 2 to 5 μm. Several reach 7 μm in size. Most of the particles in the thermo-mechanically affected zone (TMAZ) and HAZ are from 8 to 10 μm in the diameter. The shear texture rotated around the normal direction (ND)-cube texture Cube ND (001) [110] formed in the WNZ due to the stirring action of the tool head. The strongest textures in the HAZ and base material (BM) are Goss (011) [100]. The proportions of the textures in the HAZ, TMAZ, and WNZ are shown in Figure 5. They will be used in the mesoscopic models.

## 3. Joint Macroscopic Model

### 3.1. Composition of Joint Macroscopic Model

**Figure 6:**
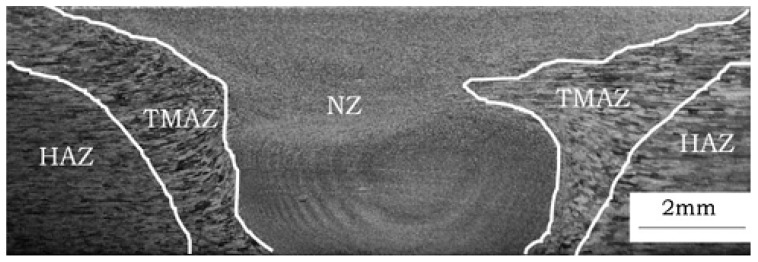
Metallographic morphology of the joint.

The joint macroscopic model that was established with ABAQUS software (6.10, Dassault system, Providence, RI, USA) is to observe the fatigue weak areas under different stress levels in the macro viewpoint. The FSW joint model is composed of WNZ, TMAZ, HAZ, and BM according to the metallurgical morphology and hardness distribution of the joint. The metallurgical morphology in the cross-section of the joint is shown in Figure 6.

### 3.2. Setting of Material Attributes

Combined isotropic/kinematic hardening model is selected for the definition of the material property. The model is composed of two components: a nonlinear kinematic hardening component and an isotropic hardening component. The kinematic hardening component is defined to be an additive combination of a linear kinematic term (Ziegler hardening law) and a relaxation term, which introduces the nonlinearity. Only the isotropic hardening model is used since the cyclic stress and strain are used to characterize the material properties.

The material properties of each zone in the FSW are set with an elasticity modulus, cyclic stress, and plastic strain data. The elastic moduli of different zones were gained from the micro-tension tests. Cyclic stress–strain data of each zone for the FSW joint were obtained with the Ramberg–Osgood equation, as listed below.

(1) Δε2=Δσ2E+(Δσ2K′)1n′

where *ε*_a_ is the strain amplitude, *σ*_a_ is the stress amplitude, *K*’ is the cyclic strength coefficient, and *n*’ is the cyclic strain hardening exponent.

The fatigue parameters of various zones were gained by the four-point correlation method proposed by Manson [21] with static mechanical property parameters that obtained from the micro-tension tests. Additionally, the cyclic strength coefficient and cyclic strain hardening exponent can be obtained by solving the following three simultaneous equations.

(2) Δσ2=K′(Δεp2)n′

(3) Δεp2=εf′(2Nf)c

(4) Δσ2=σf′(2Nf)b

where $\sigma_{f}^{\prime }$ is the fatigue strength coefficient, b is the fatigue strength exponent, $\varepsilon_{f}^{\prime }$ is the fatigue ductility coefficient, and c is the fatigue ductility exponent.

The expressions of cyclic strength coefficients and cyclic strain hardening exponents are listed below.

(5) $$K^{\prime }=\frac{\sigma_{f}^{\prime }}{\varepsilon_{f}^{\prime }{}^{n^{\prime }}}$$

(6) $$n^{\prime }=b/c$$

The calculated fatigue parameters of different zones in the welded joint are listed in Table 1.

### 3.3. Simulation Results

**Figure 7:**
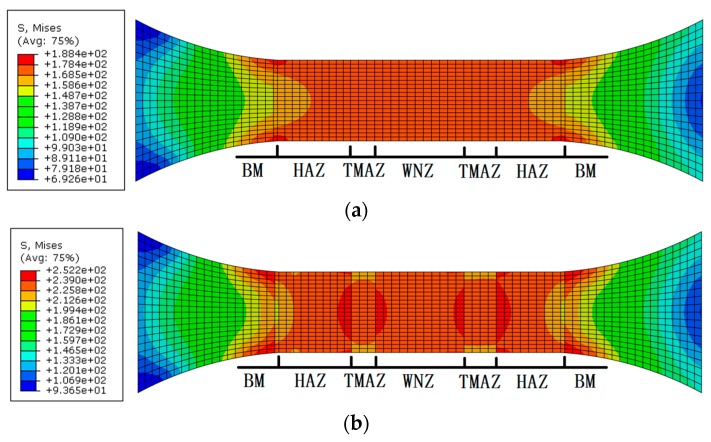

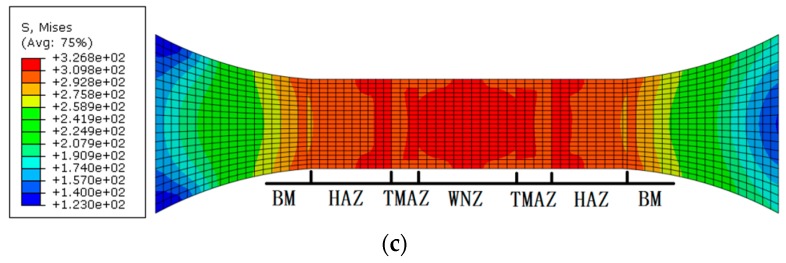
Von Mises stress distribution of the FSW joint under maximum stress of (**a**) 170 MPa, (**b**) 230 MPa, and (**c**) 300 MPa with the stress ratio of −0.3 MPa.

Four-node bilinear plane stress element CPS4R is selected to solve the structure. A total of 1626 elements and 1755 nodes are generated to simulate the FSW joints. The cyclic load of the maximum stresses of 170 MPa, 230 MPa, and 300 MPa with the stress ratio of −0.3 were applied on the joint model. The stress responses of the joint are shown in Figure 7. The maximum von Mises equivalent stresses appear at different locations under the three different stress levels. The maximum von Mises stress of 188 MPa appears at the juncture of the BM and the HAZ under the loading stress of 170 MPa. When loading stress increases to 230 MPa, the maximum von Mises stress of 245 MPa happens at the interface of the BM and the HAZ and the juncture of the HAZ and the TMAZ. The maximum von Mises stress of 327 MPa appears in the junctional area of the HAZ and the TMAZ when the loading stress is 300 MPa. The location of the stress concentration is closer to the WNZ from the BM with the rise of loading stress.

**Figure 8:**
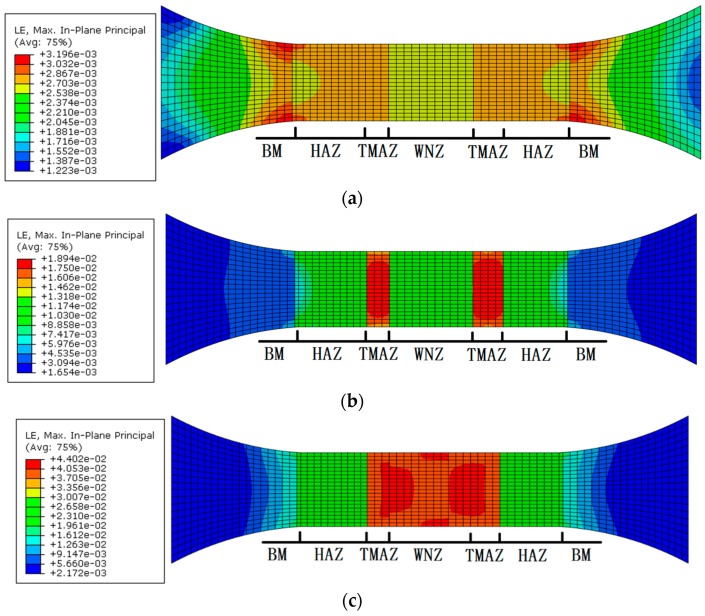
Principal strain distribution of the FSW joint under loading stress of (**a**) 170 MPa, (**b**) 230 MPa, and (**c**) 300 MPa.

The distribution of the maximum principal strain on the joint under cyclic maximum stresses of 170 MPa, 230 MPa, and 300 MPa with the stress ratio of −0.3 is shown in Figure 8. The maximum principal strain of 0.0032 is located at the interface of the BM and the HAZ when loading cyclic maximum stresses is 170 MPa. The maximum principal strain of 0.0194 appears in the TMAZ under cyclic maximum stresses of 230 MPa and the maximum principal strain value of 0.0433 happens in the junctional area of the WNZ and the TMAZ under cyclic maximum stresses of 300 MPa. The maximum strain position moves closer to the WNZ when loading stress rises.

Fatigue crack sources are mostly initiated at the WNZ and the boundary of the WNZ and the TMAZ in the fatigue experiments for the FSW joints. Some main cracks appeared in the TMAZ and the HAZ. The simulation results are basically consistent with the experimental result. However, the simulation results of the joint macroscopic model do not reflect the influence of the microstructure. Therefore, the representative volume elements (RVEs) that include the microstructure characteristics of the different zones in the joint are inserted in the joint macroscopic model as sub-models to further analyze the local stress and strain responses from a macroscale to a mesoscale.

## 4. Macro-Mesoscopic Joint Model

The sub-models inserted into the joint macroscopic model are to observe the comprehensive effect of hardening particles and grains on the local stress and strain responses. The RVEs only including grain information are also imbedded in the joint macroscopic model to compare the effect of hardening particles.

### 4.1. Mesoscopic Joint Model

**Figure 9:**
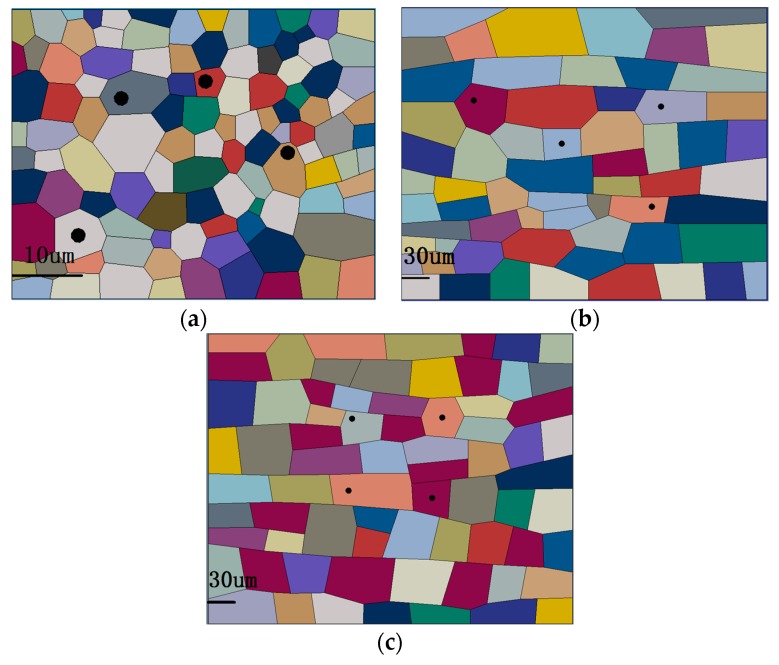
RVEs of (**a**) WNZ, (**b**) TMAZ, and (**c**) HAZ in the joint.

The mesoscopic model including characteristics of hardening particles and grains is established based on the electron backscattered diffraction (EBSD) crystal and metallurgical morphologies. The sizes of the RVEs were determined by the metallographic images. The number of grains in each RVE were calculated according to the average grain size of each zone under the same area and the voronoi polygon diagrams were generated with the voronoi function of MATLAB software (7.0, MathWorks, Natick, MA, USA). The RVEs were generated after the graphics and then they were imported into ABAQUS software, as shown in Figure 9. The morphologies of the elongated plate strip characteristics of other zones were adjusted with AUTO CAD software (2010, Autodesk, San Rafael, CA, USA). Then, the hardening particles were set in the ABAQUS software. The size and distribution of the hardening particles in each zone of the joint are different. The sizes of hardening particles are determined according to the metallography.

The crystal plasticity constitutive relation based on Huang [22] was assigned to each grain in the RVE. Random grain orientation was generated with MATLAB software and the grain orientation was input with the miller index. A material library was created with the python script language and the material properties containing grain orientation were assigned to each grain in the RVEs. The textures with specific orientation were set according to the percentage of each texture in Figure 5 and the rest of the grains were assigned with random grain orientation.

### 4.2. Crystal Plasticity Theory 

The UMAT program framework of crystal plasticity constitutive relation is referenced from Reference [22]. The Asaro hardening model is used in the constitutive law [23]. The theory of crystal plasticity is a constitutive theory based on the dislocation slip mechanism of crystal materials and mesoscopic mechanics theory. The theory regards discontinuous dislocation motion as a continuous plastic deformation process through the thought of statistics and the continuum mechanics. The plastic deformation of single crystal is attributed to the dislocation motion of the crystallographic plane and grain orientation. Self-hardening and latent hardening are introduced to describe the dislocation interaction in the slip systems in the classical crystal plasticity theory.

The tangent modulus method for a rate dependent solid developed is used in the linear solution process. The relation between shear strain $\gamma^{\left(\alpha \right)}$ and time increment Δt can be described by the following formula.

(7) $$\Delta \gamma^{\left(\alpha \right)}=\gamma^{\left(\alpha \right)}\left(t+\Delta t\right)-\gamma^{\left(\alpha \right)}\left(t\right)$$

The increments of shear strain $\Delta \gamma^{\left(\alpha \right)}$ can be obtained through Taylor expansion and consolidation.

(8) $$\Delta \gamma^{\left(\alpha \right)}\approx \Delta t\left[{\dot{\gamma }}_{t}^{\left(\alpha \right)}+\theta \frac{\partial {\dot{\gamma }}^{\left(\alpha \right)}}{\partial \tau^{\left(\alpha \right)}}\Delta \tau^{\left(\alpha \right)}+\theta \frac{\partial {\dot{\gamma }}^{\left(\alpha \right)}}{\partial g^{\left(\alpha \right)}}\Delta g^{\left(\alpha \right)}\right]$$

where ${\dot{\gamma }}_{t}^{\left(\alpha \right)}$ is the slipping rate, $\Delta \tau^{\left(\alpha \right)}$ is the increments of resolved shear stress, $\Delta g^{\left(\alpha \right)}$ is the increments current strength, and the parameter θ ranges from 0 to 1.

The symmetric part w and the asymmetrical part μ of the schmid tensor of the slip system are introduced below.

(9) $$\mu_{\mathrm{ij}}^{\left(\alpha \right)}=\frac{1}{2}\left(s_{i}^{\left(\alpha \right)}m_{j}^{\left(\alpha \right)}+s_{j}^{\left(\alpha \right)}m_{i}^{\left(\alpha \right)}\right)$$

(10) $$w_{\mathrm{ij}}^{\left(\alpha \right)}=\frac{1}{2}\left(s_{i}^{\left(\alpha \right)}m_{j}^{\left(\alpha \right)}-s_{j}^{\left(\alpha \right)}m_{i}^{\left(\alpha \right)}\right)$$

where unit vectors $s^{\left(\alpha \right)}$ and $m^{\left(\alpha \right)}$ are the slip direction and normal to slip plane in the reference configuration, respectively.

Increments current strength $\Delta g^{\left(\alpha \right)}$, the increments of resolved shear stress $\Delta \tau^{\left(\alpha \right)}$, and corotational stress increments $\Delta \sigma_{\mathrm{ij}}$ can be described with the following formula.

(11) $$\Delta g^{\left(\alpha \right)}=\underset{\beta }{\sum }h_{\alpha \beta }\Delta \gamma^{\left(\beta \right)}$$

(12) $$\Delta \tau^{\left(\alpha \right)}=\left[L_{\mathrm{ijkl}}\mu_{\mathrm{kl}}^{\left(\alpha \right)}+w_{\mathrm{ik}}^{\left(\alpha \right)}\sigma_{\mathrm{jk}}+w_{\mathrm{jk}}^{\left(\alpha \right)}\sigma_{\mathrm{ik}}\right]\Delta \left[\Delta \varepsilon_{\mathrm{ij}}-\underset{\beta }{\sum }\mu_{\mathrm{ij}}^{\left(\beta \right)}\Delta \gamma^{\left(\beta \right)}\right]$$

(13) $$\Delta \sigma_{\mathrm{ij}}=L_{\mathrm{ijkl}}\Delta \varepsilon_{\mathrm{kl}}-\sigma_{\mathrm{ij}}\Delta \varepsilon_{\mathrm{kk}}-\underset{\beta }{\sum }\left[L_{\mathrm{ijkl}}\mu_{\mathrm{kl}}^{\left(\alpha \right)}+w_{\mathrm{ik}}^{\left(\alpha \right)}\sigma_{\mathrm{jk}}+w_{\mathrm{jk}}^{\left(\alpha \right)}\sigma_{\mathrm{ik}}\right]$$

where $h_{\alpha \beta }$ are the slip hardening moduli, $\Delta \varepsilon_{\mathrm{ij}}$ are strain increments, and $L_{\mathrm{ijkl}}$ are the elastic moduli. 

For a particular strain increment $\Delta \varepsilon_{\mathrm{ij}}$, the shear strain increment can be determined by the following formula.

(14) $$\underset{\beta }{\sum }\{\delta_{\alpha \beta }+\theta \Delta t\frac{\partial {\dot{\gamma }}^{\left(\alpha \right)}}{\partial \tau^{\left(\alpha \right)}}\left[L_{\mathrm{ijkl}}\mu_{\mathrm{kl}}^{\left(\alpha \right)}+w_{\mathrm{ik}}^{\left(\alpha \right)}\sigma_{\mathrm{jk}}+w_{\mathrm{jk}}^{\left(\alpha \right)}\sigma_{\mathrm{ik}}\right]\mu_{\mathrm{ij}}^{\left(\beta \right)}-\theta \Delta t\frac{\partial {\dot{\gamma }}^{\left(\alpha \right)}}{\partial g^{\left(\alpha \right)}}h_{\alpha \beta }\mathrm{sign}\left({\dot{\gamma }}_{t}^{\left(\beta \right)}\right)\}\Delta \gamma^{\left(\beta \right)}\;={\dot{\gamma }}_{t}^{\left(\alpha \right)}\Delta t+\theta \Delta t\frac{\partial {\dot{\gamma }}^{\left(\alpha \right)}}{\partial \tau^{\left(\alpha \right)}}\left[L_{\mathrm{ijkl}}\mu_{\mathrm{kl}}^{\left(\alpha \right)}+w_{\mathrm{ik}}^{\left(\alpha \right)}\sigma_{\mathrm{jk}}+w_{\mathrm{jk}}^{\left(\alpha \right)}\sigma_{\mathrm{ik}}\right]\Delta \varepsilon_{\mathrm{ij}}$$

where $\delta_{\alpha \beta }$ is the Kronecker delta.

The shear strain is solved with the following nonlinear equations.

(15) $$\Delta \gamma^{\left(\alpha \right)}-\left(1-\theta \right){\dot{\gamma }}_{t}^{\left(\alpha \right)}\Delta t-\theta \Delta {t\dot{a}}^{\left(\alpha \right)}f^{\left(\alpha \right)}\left(\frac{\tau_{t}^{\left(\alpha \right)}+\Delta \tau^{\left(\alpha \right)}}{g_{t}^{\left(\alpha \right)}+\Delta g^{\left(\alpha \right)}}\right)=0$$

where the constant ${\dot{a}}^{\left(\alpha \right)}$ is the reference strain rate on slip system α while $g_{t}^{\left(\alpha \right)}$ is a variable that describes the current strength of that system, $\tau_{t}^{\left(\alpha \right)}$ is the resolved shear stress, and $f^{\left(\alpha \right)}$ is a general function describing the dependence of the strain rate on the stress.

### 4.3. Material Attributes

The RVEs containing crystal characteristics and hardening particles of the WNZ, TMAZ, and HAZ were inserted into the central edge of the corresponding zones in the above macro FSW joint model. To prevent the severe mesh distortion, the transition regions are set outside the RVEs to mesh with different sizes. Material attributes outside RVEs are input in the same way as that in the above joint macroscopic model and the material attributes inside RVEs are input as follows.

The anisotropic cubic elastic constants of grains in the crystal plasticity method are set to be C11 = 106750 MPa, C12 = 60410 MPa, and C44 = 28300 MPa. The strain rate sensitivity factor n is 20 and the reference shear strain rate ${\dot{\gamma }}^{0}$ is 0.001 [24]. Other parameters of different zones are determined by the try and error method [20]. 

The hardening particles are simplified to be isotropic. The hardness of hardening particles and matrix and elastic modulus of some large size hardening particles were measured with Agilent Nano Indenter G200. The elastic modulus of hardening particles is 95775 MPa, which is the average value of 10-point measurements from a different area. The mixing rules were used for the stress and plastic strain values of the hardening particles with the area fractions, the hardness of the matrix, and the hardening particles, as listed below [25,26]. The area fractions were obtained from the metallurgic morphology with Photoshop software.

(16) $$\sigma (\varepsilon_{p})=(1-S_{f}^{\beta })\sigma_{\alpha }(\varepsilon_{p})+S_{f}^{\beta }\sigma_{\beta }(\varepsilon_{p})$$

(17) $$\frac{\sigma_{\alpha }(\varepsilon_{p})}{\sigma_{\beta }(\varepsilon_{p})}=\frac{H_{\alpha }}{H_{\beta }}$$

where $\sigma \left(\varepsilon_{p}\right)$ is total equivalent stress as a function of equivalent plastic strain, $\sigma_{\alpha }\left(\varepsilon_{p}\right)$ is the equivalent stress of the matrix as a function of equivalent plastic strain, $\sigma_{\beta }\left(\varepsilon_{p}\right)$ is the equivalent stress of the hardening phase as a function of equivalent plastic strain, $S_{f}^{\beta }$ is the area fraction of the hardening phase, $H_{\alpha }$ is the Vickers hardness of the matrix, and $H_{\beta }$ is the Vickers hardness of the hardening phase. 

The stress and plastic strain data for the hardening particles were input into the setting of material attribute with the selection of combined isotropic/kinematic hardening model for the plasticity.

## 5. Simulation

### 5.1. Responses of Stress and Strain

**Figure 10:**
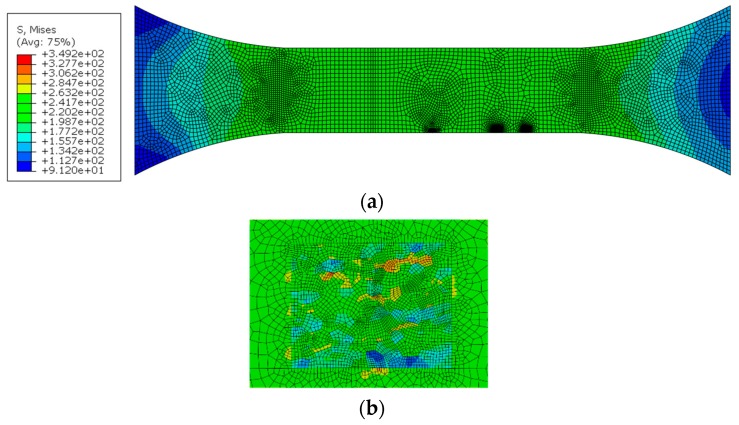
(**a**) Von Mises stress nephogram in the macro-mesoscopic joint model, (**b**) Von Mises stress distribution in the WNZ, MPa.

Four-node bilinear element CPS4R is used in the mesoscopic model, which is the same with that in the macroscopic model. The boundary constrain condition was added at one side and the cyclic stress of 230 MPa with a stress ratio of −0.3 was applied to the other side along the axial direction in the joint model. The stress response of the joint macro-mesoscopic model under the maximum stress of 230 MPa with a stress ratio of −0.3 is shown in Figure 10. The stress values are much greater than those in the joint macroscopic model. The maximum von Mises stress and principal strain appears at the WNZ. No stress concentration happens in the border of RVE, as shown in Figure 10b. It suggests that it is suitable for the setting of the transition region.

**Figure 11:**
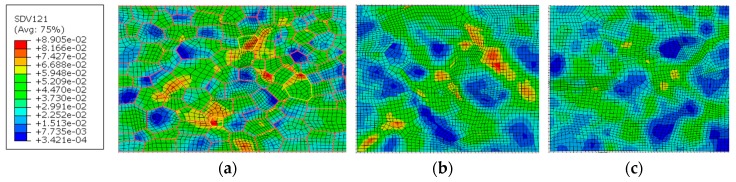
Cumulative shear strains of all slip systems: (**a**) WNZ, (**b**) TMAZ, and (**c**) HAZ.

The cumulative shear strains of all slip systems in Figure 11 show that the distribution of the shear strains is nonuniform in these zones. Grain orientation in the WNZ is most favorable for the slip systems. Most grains deform and larger strains happen in multiple areas in the WNZ, which is followed by the TMAZ. The least activation of the slip system is in the HAZ. The maximum shear strain in the WNZ is close to that in the TMAZ. Fatigue weak areas can be deduced so that it should be the WNZ, and then the TMAZ under the current loading condition in the cumulative shear strain analysis. 

The larger shear strains in the WNZ were analyzed from the viewpoint whether it is related to the texture orientation. Three grains, where their boundaries are marked yellow in Figure 11a, have the maximum or close to the maximum strains in the WNZ. Other grain boundaries are marked in red. The analysis results show that larger strains happen in the grain boundaries and the orientation of these grains is random instead of the specific texture.

### 5.2. Misorientation Analysis

**Figure 12:**
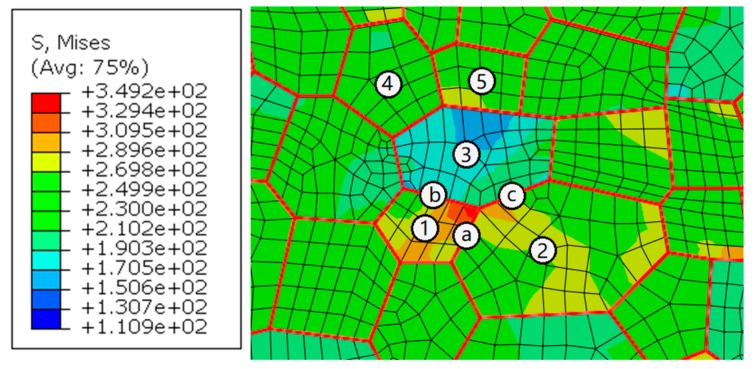
Stress concentration at the grain boundary marked as letter a, MPa.

The influence of misorientation is analyzed by selecting a group of grains from the site with a stress concentration in the WNZ. As shown in Figure 12, numbers and letters represent grains and grain boundaries. All grain boundaries are marked in red to better identify them. The misorientations of adjacent grains are extracted and listed in Table 2. Only the misorientation angle between grains Nos. 1 and 2 is low. The misorientation angles of other adjacent grains are high. The stress concentration transmits to the neighbor grain No. 2 through a small-angle misorientation. However, the boundaries marked as letters b and c hinder the stress transfer because of their high-angle misorientation. The stress in the grain No. 3 keeps a low level. The stress concentration only happens near the grain boundary marked as letter a.

**Figure 13:**
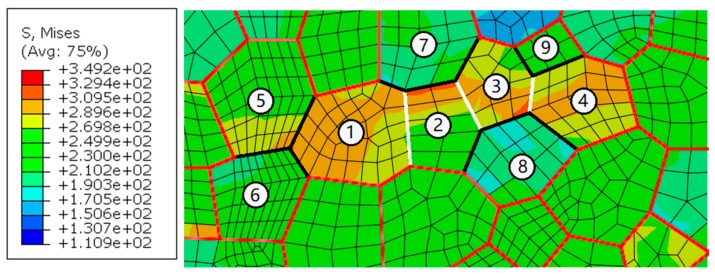
Stress transfer through low-angle misorientation grain boundaries, MPa.

Another group of grains having stress concentrations is selected to further verify the influence of misorientation. The misorientations of adjacent grains are listed in Table 3. In Figure 13, the black and white line segments stand for the high-angle and low-angle misorientations of adjacent grains, respectively. It is apparent that the stress transfer through the grain boundaries have low-angle misorientation. The high-angle misorientation of adjacent grains has a blockade for the stress transfer and hinder the crack propagation [27].

### 5.3. Effect of Hardening Particles

The RVEs including the crystal characteristic and hardening particles, and only containing the crystal characteristic, were embedded into the macroscopic model, respectively. The diameter of hardening particles in the WNZ is set at 2 μm and the hardening particles in the HAZ and TMAZ are set as 8 μm in diameter in the RVEs.

The distribution of stress and strain in different zones from the mesoscopic point of view is quite different. The maximum stress in the RVE of each zone is significantly higher than that in the joint macroscopic model. The maximum stress of 349 MPa appears at the junction of three grains in the WNZ, while the macroscopic von Mises stresses are mainly between 230 MPa to 250 MPa. The maximum strain of 0.0384 also occurs at the grain boundary of the WNZ in the macro-mesoscopic joint model, while the macroscopic strain is about 0.012.

**Figure 14:**
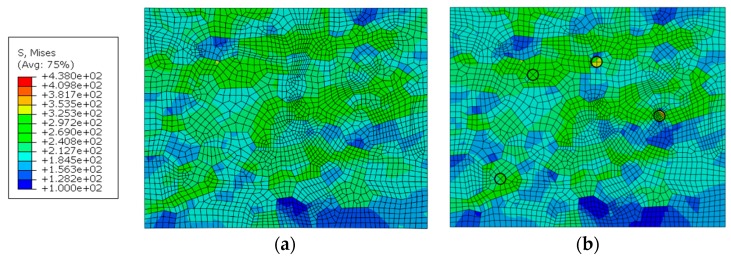
Von Mises stress distribution of WNZ in the joint models that, (**a**) only containing crystal characteristics, and (**b**) including crystal characteristics and hardening particles MPa.

**Figure 15:**
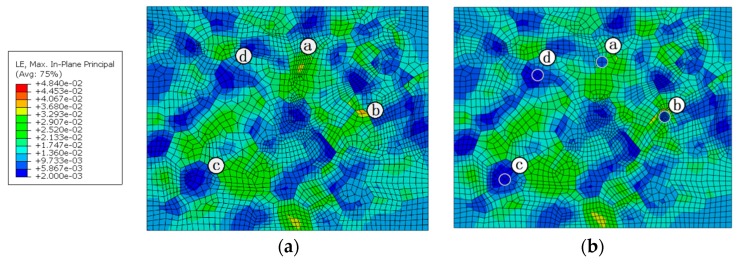
Maximum principal strain distribution of WNZ in the joint models that (**a**) only contain crystal characteristics, and (**b**) include crystal characteristics and hardening particles.

The effect of hardening particles on the stress and strain responses can be seen from Figure 14 and Figure 15. It can be seen that the existence of the hardening particles causes the increase of the stress and strain around the hardening particles. The emergence of the hardening particles will further raise the strain in the location of strain concentration. The maximum strain values of the points a and b in the WNZ of Figure 15a increase from 0.0338 and 0.0384 to 0.0347 and 0.0484, respectively, when the hardening particles appear. The maximum stress and strain in each zone are shown in Table 4 and Table 5. The stress/strain concentration value is defined as the ratio of maximum von Mises stress/principal strain at the grain boundary for material including particles to material without particles. The stress concentration value in the WNZ is not large but the strain concentration value is the largest in the three zones. Stress concentrations around points a and b are highlighted due to the hardening particles. However, the stresses and strains around the hardening particles, where the strains are small, as shown in points c and d in Figure 15b, do not show a clear change by comparison among Figure 14a and Figure 15a.

### 5.4. Size of the Hardening Particle

**Figure 16:**
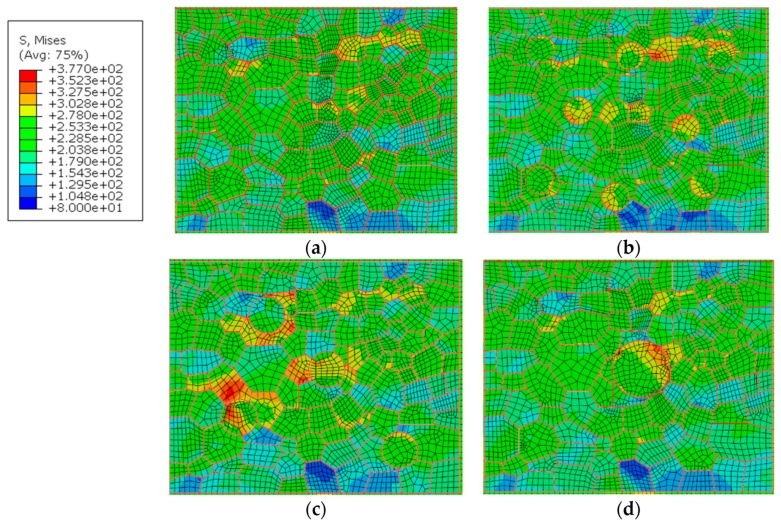
Stress distribution (**a**) without particle, and with (**b**) 5μm *8, (**c**) 6.3μm *4, and (**d**) 10μm *1 particles, MPa.

**Figure 17:**
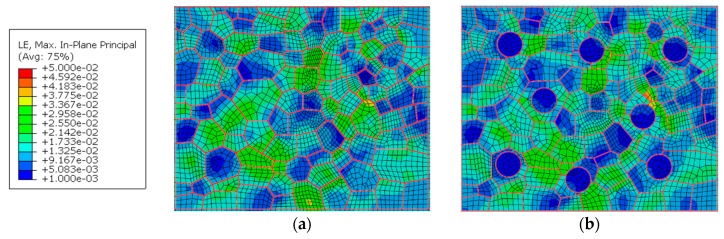

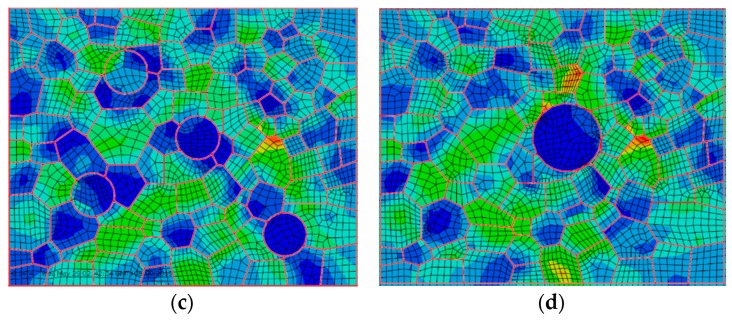
Strain distribution (**a**) without particle, and with (**b**) 5μm *8, (**c**) 6.3μm *4, and (**d**) 10μm *1 particles.

For studying the effect of the hardening particle size on the local mechanical properties of the joint, different sizes of hardening particles with the same volume fraction were added into the RVE of the WNZ. The hardening particles were placed in the location away from the border of the RVE. The diameter and numbers of the particles were set at 5μm*8, 6.3μm*4, and 10μm*1, as shown in Figure 16b–d. The setting of material property is the same as above. The maximum cyclic stress of 230 MPa with a stress ratio of −0.3 was applied to the model. The distribution of stresses and strains in the WNZ is shown in Figure 16 and Figure 17. The stresses and strains around the particles are affected by the sizes and numbers of the hardening particles and the stress and strain distribution is different around the same size particles. This means they may be influenced by the grain boundary and grain orientation. All grain boundaries are marked in red in Figure 16 and Figure 17. Maximum stress in the WNZ changes from 349 to 344, 376, and 339 MPa and the maximum strain increases from 0.038 to 0.045, 0.046, and 0.050. The strains around the particles show a growth trend with the rise of the particle size. The stress or strain concentrations are affected by the size and distribution of hardening particles, grain boundaries, and grain orientation [28].

### 5.5. Hardening Particle Clustering

**Figure 18:**
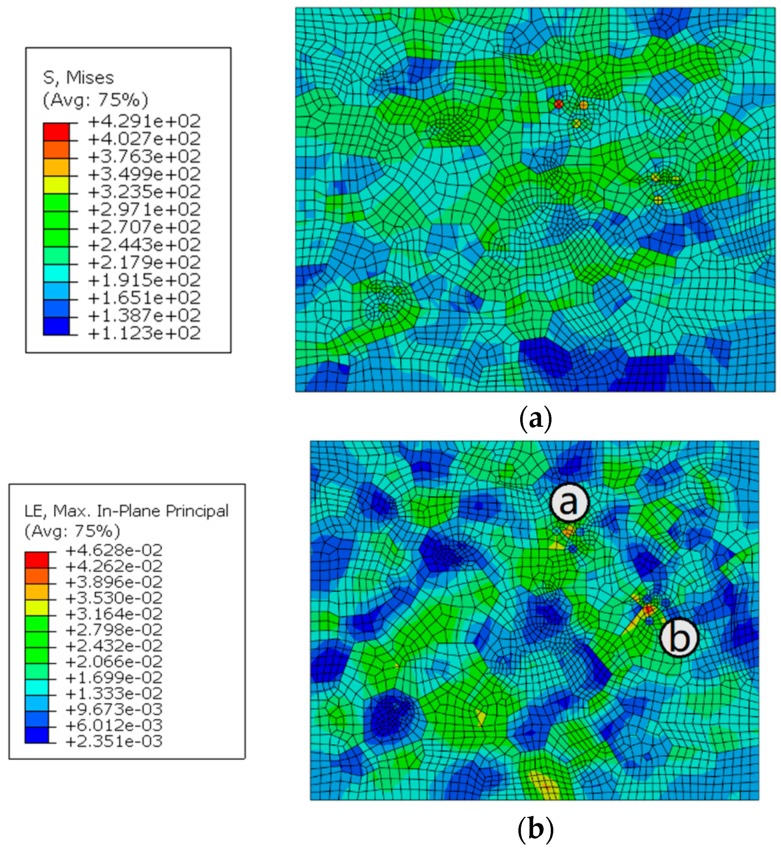
Distribution of (**a**) von Mises equivalent stress, MPa, and (**b**) principal strain in the WNZ including the hardening particle cluster.

In order to study the effect of the clustering particles on the mechanical property of joint, several small hardening particles with the diameter of 1 μm were inserted into the RVE of the WNZ. The distribution of stress and strain in the WNZ under the maximum stress of 230 MPa with a stress ratio of −0.3 is shown in Figure 18. Maximum strains near points a and b reach to 0.0386 and 0.0463. The strain concentration caused by multiple particles is significantly higher than that of single hardening particle with the same size. It indicates that the gathering of hardening particles also leads to a strain concentration and likely produces fatigue cracks, which are in accordance with the analysis for the fracture morphology of Figure 3.

### 5.6. Cracked Hardening Particle

**Figure 19:**
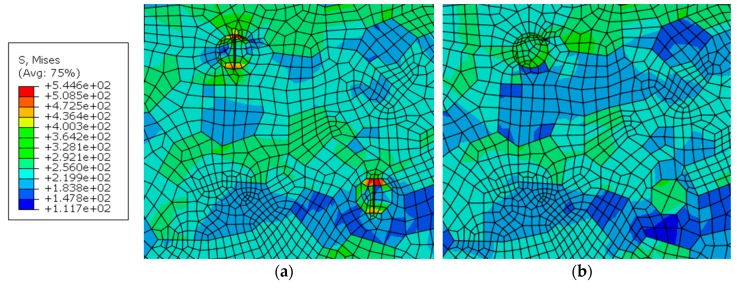
Distribution of von Mises equivalent stress in the WNZ of including (**a**) cracked particles and (**b**) uncracked particles, MPa.

**Figure 20:**
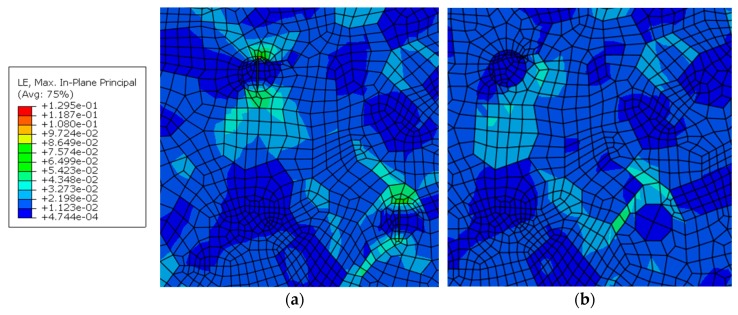
Distribution of principal strain in the WNZ including (**a**) cracked particles and (**b**) uncracked particles.

The effects of cracked particles on the stress and strain are conducted by comparison with the uncracked particles. The stresses and strains at the tip of cracked particles have a substantial increase. The maximum stress at the crack tip of the particles is 545 MPa in Figure 19 and it has a clear increase by comparison with the stress of 327 MPa on the uncracked particle in the same size at the same site. The strain distribution around the crack tip of the particles is shown in Figure 20. The strain value is 0.130, which is much larger than 0.045 around the uncracked particle. The concentration of the stress and strain at the crack tip is one of the causes of the crack initiation and growth.

## 6. Conclusions

(1) The joint macroscopic model is composed of different zones that have different properties. The simulation shows that the fatigue weak area is gradually close to the WNZ from the BM with an increase of loading stress.
(2) The macro-mesoscopic joint model containing the hardening particles and the crystal characteristics is established with different settings for the material properties of particles and grains.
(3) The responses of local stress and strain in the WNZ are analyzed from the status of the slip system activation, grain misorientation, particle size, particle cluster, and cracked particles. The stress transfer occurs at the grain boundaries with low-angle misorientations. Clustering of hardening particles is prone to cause the strain increase and the cracked particles also make the local stress and strain an apparent rise.

## Figures and Tables

**Table 1:** Fatigue parameters of the welded joint.

Zones	Elastic modulus *E* (GPa)	Fatigue Strength Coefficient *σ*’_f_ (MPa)	Fatigue Strength Exponent *b*	Fatigue Ductility Exponent *c*	Fatigue Ductility Coefficient *ε*’_f_	Cyclic Strength Coefficient *K*’(MPa)	Cyclic Strain Hardening Exponent *n*’
BM	57	514	−0.089	−0.465	0.098	801	0.192
HAZ	62	432	−0.089	−0.425	0.093	713	0.211
TMAZ	65	424	−0.094	−0.43	0.105	694	0.219
WNZ	66	424	−0.094	−0.441	0.118	668	0.213

**Table 2:** The misorientations of adjacent grains in Figure 12.

**Adjacent grains**	1,2	1,3	2,3	3,4	3,5	4,5
**Misorientation/°**	3	45	42	29	45	41

**Table 3:** Misorientations of adjacent grains in Figure 13.

**Adjacent grains**	1,2	2,3	3,4	5,6	1,5	1,6	1,7	2,7	2,8	3,8	3,9	4,8	4,9
**Misorientation/°**	11	17	9	48	35	43	52	44	41	55	43	49	35

**Table 4:** Maximum von Mises stress of RVE in each zone (MPa).

Zone	WNZ	TMAZ	HAZ
**Without particles**	349	310	339
**Including particles**	378	402	437
**Stress concentration value**	1.09	1.30	1.29

**Table 5:** Maximum principal strain of RVE in each zone.

Zone	WNZ	TMAZ	HAZ
**Without particles**	0.0384	0.0363	0.0322
**Including particles**	0.0484	0.0375	0.0324
**Strain concentration value**	1.26	1.03	1.01

## Abbreviations

FSW	Friction stir welding
WNZ	Weld nugget zone
TMAZ	Thermo-mechanically affected zone
HAZ	Heat affect zone
BM	Base material
SEM	Scanning electron microscope
ND	Normal direction
RVE	Representative volume element
